# The causes of spatiotemporal variations in erupted fluxes and compositions along a volcanic arc

**DOI:** 10.1038/s41467-019-09113-0

**Published:** 2019-03-22

**Authors:** C. B. Till, A. J. R. Kent, G. A. Abers, H. A. Janiszewski, J. B. Gaherty, B. W. Pitcher

**Affiliations:** 1 School of Earth & Space Exploration, Arizona State University, Tempe, AZ USA; 2 College of Earth, Ocean, & Atmospheric Sciences, Oregon State University, Corvallis, OR USA; 3 Department of Earth & Atmospheric Sciences, Cornell University, Ithaca, NY USA; 4 Lamont Doherty Earth Observatory, Columbia University, New York, NY USA; 5 Present Address: DTM, Carnegie Institution for Science, Washington, DC 20015 USA; 6 Present Address: Earth and Environmental Sciences, Vanderbilt University, Nashville, TN 37240 USA

## Abstract

Decades of study on volcanic arcs have provided insight into the overarching processes that control magmatism, and how these processes manifest at individual volcanoes. However, the causes of ubiquitous and dramatic intra-arc variations in volcanic flux and composition remain largely unresolved. Investigating such arc-scale issues requires greater quantitative comparison of geophysical and geochemical data, linked through sets of common intensive variables. To work towards these goals, we use observed lava compositions to estimate the heat budget associated with Quaternary volcanism in the Cascades Arc and compare this to the heat required to produce the observed geophysical properties of the crust. Here we show that along-strike volcanic variability in the Quaternary Cascades Arc is primarily related to variations in the flux of basalt into the crust, rather than variations in their crustal storage history. This approach shows promise for studying other large-scale frontier geologic problems in volcanic arcs.

## Abstract (web summary)

The primary causes of dramatic variations in volcanic flux and composition along strike in subduction zones remain largely unknown. Here we use a promising new approach to show that along-strike volcanic variability in the Quaternary Cascades Arc is primarily due to variations in the flux of basalt into the base of the crust, rather than crustal magma storage.

## Introduction

The critical importance of volcanic arcs in the formation and evolution of the continental crust and the cycling of elements through time between the crust, mantle, and exosphere has been recognized for several decades^1–5^. As a result, the overarching igneous and metamorphic processes that give rise to arc magmatism have been well studied and are, for the most part, quite successful at explaining the various magmatic compositions erupted at volcanic arcs^6–11^.

**Fig. 1:**
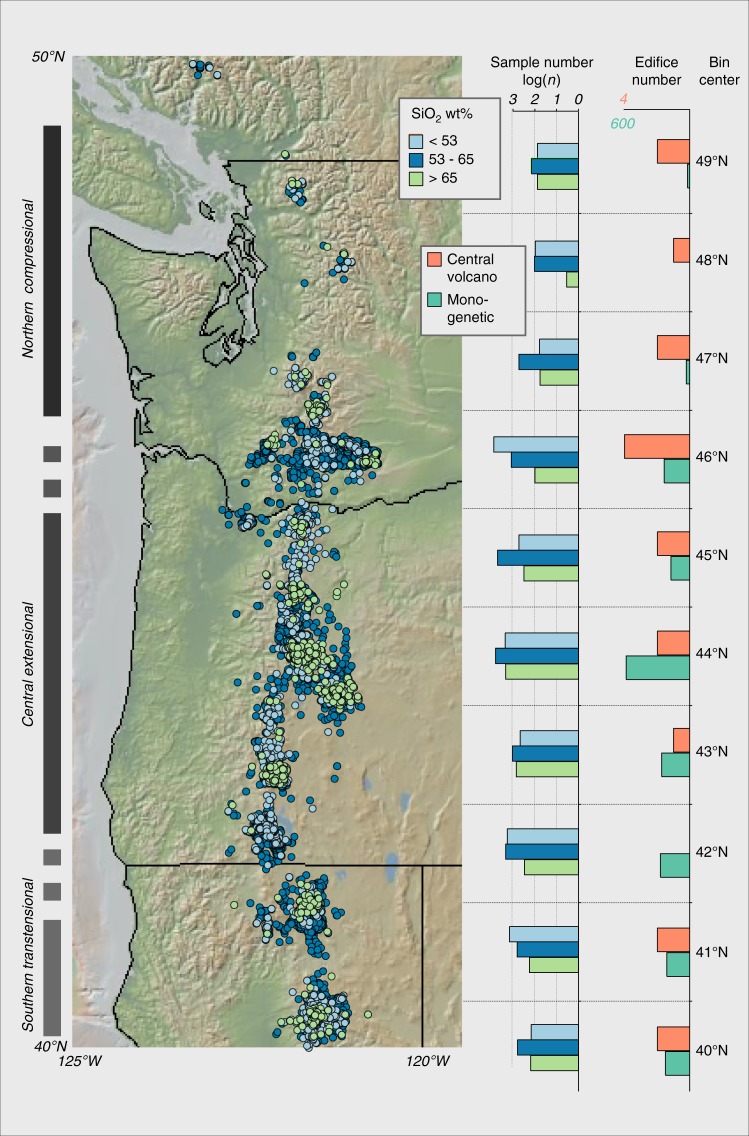
Map of Cascades Arc showing locations of geochemical samples and selected summaries of the existing datasets. The map and the accompanying histograms illustrate the available Quaternary Cascade volcanic rock compositions from Earthref.org separated into three categories according to SiO_2_ content (colors are the same for map symbols), the number of major edifices, and monogenetic vents from Hildreth^15^

However, there are other important aspects of arc volcanism that are quite poorly explained by existing models. One notable example is the dramatic diversity evident in volcanic activity within the same volcanic arc. This diversity is expressed as, among other things, large variations in erupted compositions, erupted volumes, edifice numbers, eruption style, and morphology between individual volcanoes and subarc segments—and even between volcanoes that lie in close spatial association^8,12–17^. In the Cascades Arc, for example, erupted volumes differ by a factor of two between the southern and northern portions of the arc, and there are major changes in the partitioning of volcanism between intermediate and silicic-dominated central volcanic edifices and fields of more mafic and dispersed monogenetic centers (Fig. 1). Similarly, there are large variations in the geophysical state of the crust observed along strike in many arcs, including crustal and upper mantle seismic velocities and measured heat flow^18–20^, and it is unclear how these relate to variations in magmatic activity.

Intra-arc volcanic diversity also contrasts with the modern concept of subduction as a continuous or quasi-continuous process, with progressive devolatilization in the descending slab continuously producing magma with the mantle wedge. Likewise, the subduction parameters (slab age, slab dip, convergence rate and obliquity, and crustal thickness) that ultimately control the thermal state of a subduction zone typically change gradually along strike^21,22^. Thus, understanding how subduction can produce a volcanic record that is highly episodic and heterogeneous in space and time is an extant grand challenge for subduction science. Furthermore, resolving the ultimate controls over the causes of volcanic and magmatic diversity in arcs has important ramifications for understanding the dynamics of continental margins, mantle flow behavior, and volcanic hazards.

**Fig. 2:**
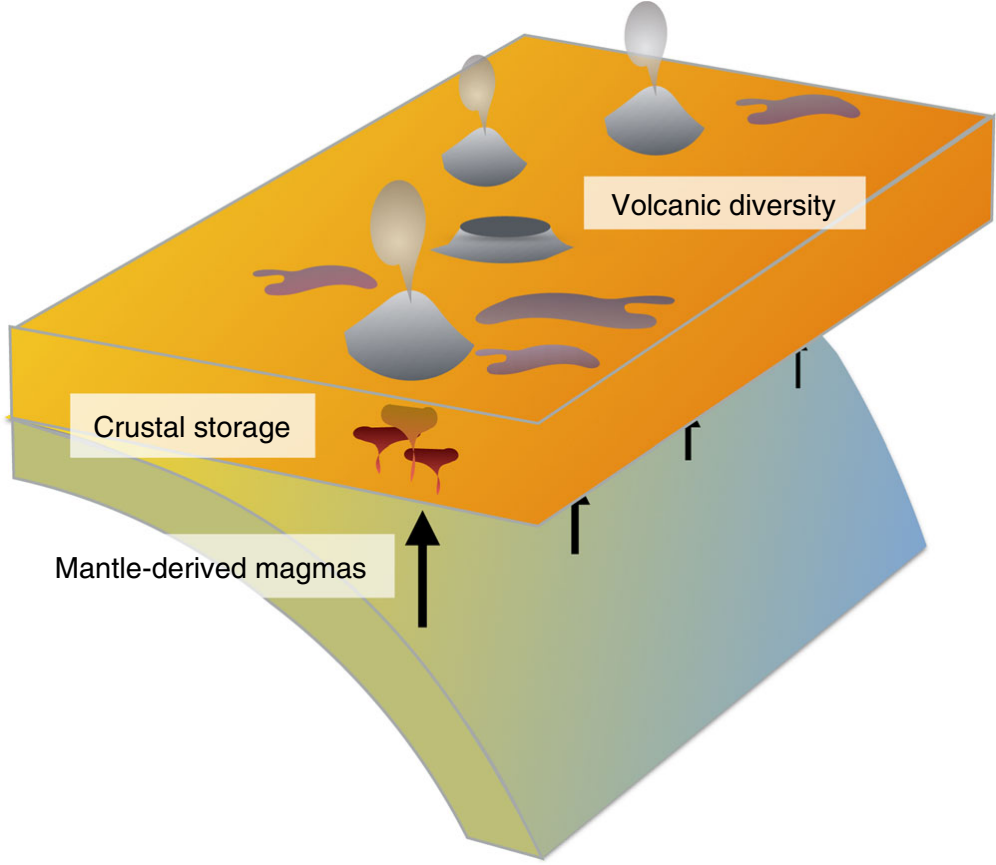
Schematic figure of the possible mantle and crustal roles in forming arc volcanic diversity. Mantle-derived magmas are input into the crust, where they are stored, crystallize, and trigger crustal melting, which in some combination produces the variation in eruptive behavior (eruptive style, composition, and flux) observed along striking a volcanic arc

We believe that the potential causes of volcanic diversity in arcs can be framed as two end-member hypotheses (Fig. 2). In the first, the observed variations in arc volcanism are produced solely by differences in crustal processes and properties, with constant mantle flux along strike^8,12,23,24^. Alternatively, variations in volcanic fluxes and compositions are primarily the result of differences in the underlying mantle flux (controlled in turn by variations in the downgoing slab and/or mantle wedge), with processes within the arc crust playing only a secondary role^13,17,25–27^. These hypotheses, used either explicitly or implicitly, have been in place for decades, but there remains little clear consensus about the relative contribution of the crust and mantle in producing volcanic diversity.

**Fig. 3:**
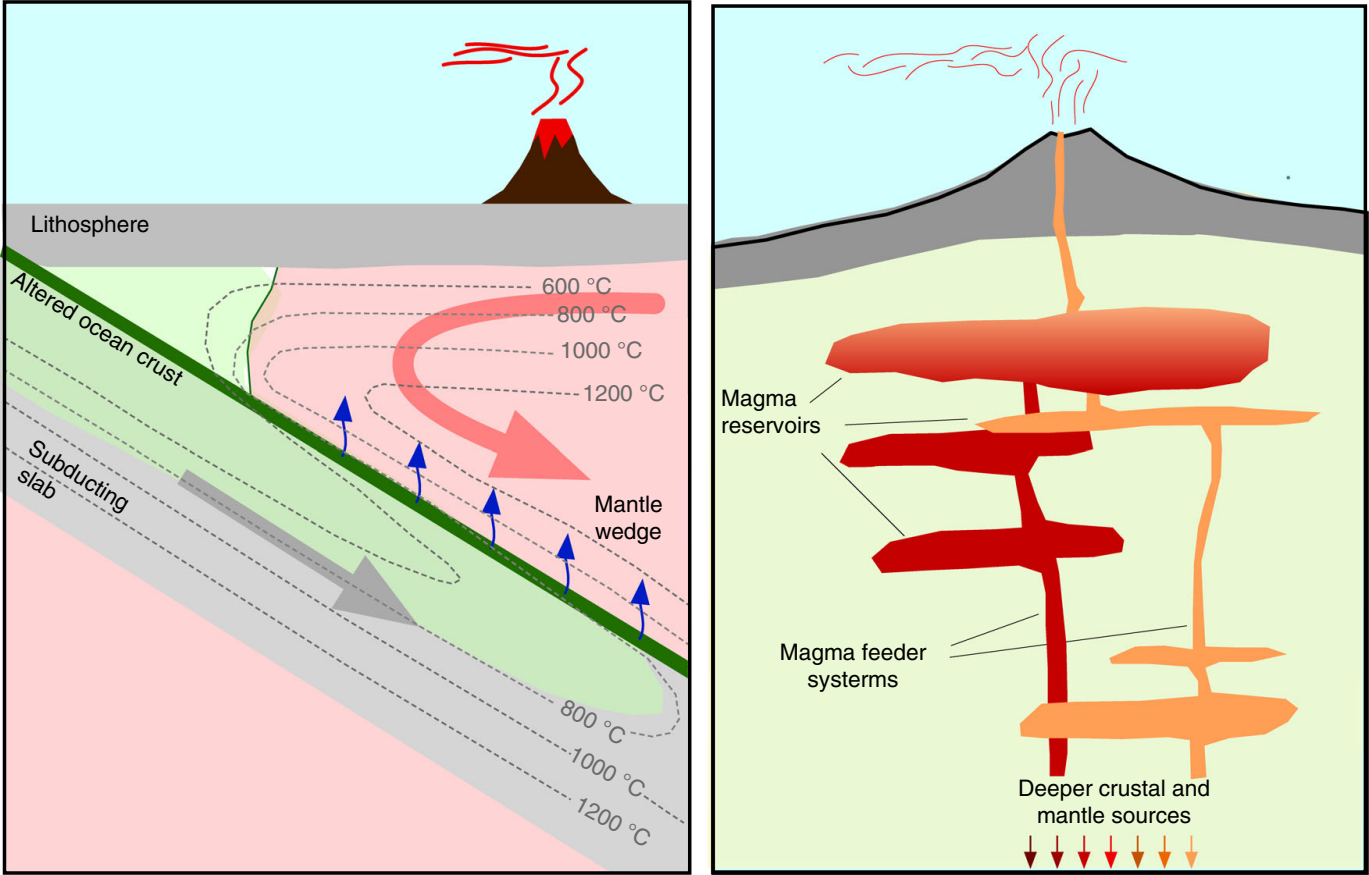
Representative figures from different communities studying volcanoes. Left, archetypical mantle-focused subduction cartoon with an “emoji volcano” that only alludes to a shallower magmatic system. Right, archetypical upper crust-focused subduction volcano cartoon, with a “disembodied volcano”, disconnected from the deeper magma source region

Resolving the importance of crust and mantle processes is clearly important to understanding arc-scale volcanic diversity. However, the existing methods for studying arc volcanism, dominated by detailed studies of individual volcanoes, also have limitations for addressing such arc-scale questions. For a start, much of what we know about arc volcanism is heavily leveraged by the intensive study of a relatively small number of volcanic systems. Although detailed studies of individual volcanoes work well for understanding the local-scale variations in magma compositions that characterize individual volcanic systems^28–33^, it is less clear how readily detailed petrologic models developed for well-studied individual volcanoes, such as Mount St. Helens and Soufrière Hills, can be ported to other parts of the systems that are less well known^34^. Moreover, geochemical sampling of arc volcanics is strongly biased by these locations of intense interest, such as well exposed or recently active volcanoes, and geochemical sampling programs are almost never designed to produce ergodic or representative datasets of erupted compositions through space and time. The result is that geochemical and petrological datasets are hard to reconcile and compare with the regional and integrative datasets produced by many geophysical studies, and this leads to qualitative, rather than quantitative comparisons^19,25,35–38^. Finally, understanding of the larger-scale controls on volcanism and magmatism is also limited by the common approach of focusing on only one part of the subduction system (e.g., upper crust or mantle wedge), with very limited consideration of the feedbacks and interactions that occur across the entire arc system. This is exemplified by the summary cartoon figures that classically accompany papers from the relevant scientific communities (including those we have written ourselves), with “disembodied volcanoes” missing the lower half of the magmatic system in the volcanology literature, or oversized “emoji volcanoes” that only allude to a shallower magmatic system in mantle-oriented petrologic studies (Fig. 3).

Here, we propose a multidisciplinary approach that strives to transcend some of these limitations. We use this approach to address the causes of intra-arc volcanic diversity in the Cascades, but we believe that the method can also be applied to other frontier questions in subduction zone science. The approach utilizes our basic understanding of the physical processes involved in volcanic arc formation to find intensive variables, such as specific heat and density, which can be used to quantitatively link and compare large-scale datasets from different disciplines. We also incorporate statistical techniques to minimize the effects of nonrepresentative geochemical sampling. To illustrate this approach, we present a case study focused on the causes of intra-arc volcanic diversity in the Cascades Arc. By focusing on the thermal effects of magmatism on the crust, we are able to compare large-scale geophysical properties (seismic velocities and surface heat flow) with observed volcanic volumes and geochemistry. Our approach suggests that the mantle is the primary driver of volcanic arc diversity in the Cascades.

## The relationship between magmatism and crustal physical properties in the Cascades Arc

We can potentially link the observed volcanic record in a volcanic arc with regional geophysical datasets by exploring the thermal effects of magmatism on the crust and mantle. Magmatism plays an important role in establishing the composition and architecture of the crust in subduction zones via addition of mass and heat, as well as crustal melting, metamorphism, and hydrothermal alteration^8–10,39–41^. These processes will in turn influence the broad-scale geophysical properties of the crust, such as surface heat flow and seismic velocities, which can then be used to constrain broad-scale magmatic processes. In order to relate the magmatic and geophysical properties of the Cascades Arc, we compare a calculated Quaternary magmatic heat budget directly with measured heat flow and the observed seismic velocities sampling a range of crustal depths. Seismic velocities and surface heat flow represent current snapshots of the state of the Cascades Arc, whereas we have used data for volcanic rocks of Quaternary age (<~2.6 Ma).

Seismic proxies for subsurface temperature provide samples of the modern geotherm, surface heat flow measures transients after they conduct through the lithosphere (on the order of 10 Ma for a 30–40-km thickness), and the volcanic record provides a Quaternary average of temperature at depths where melting occurs. All three of these observations show a close spatial correlation, despite the widely differing time ranges over which they average, implying that transient or time-evolving processes are minor over these timescales. Thus, we assume that the arc as a whole has been at a steady state in terms of the thermal influence of magmatism on the crust during the Quaternary, although clearly there are perturbations at the scale of individual volcanoes represented by episodic eruptions.

**Fig. 4:**
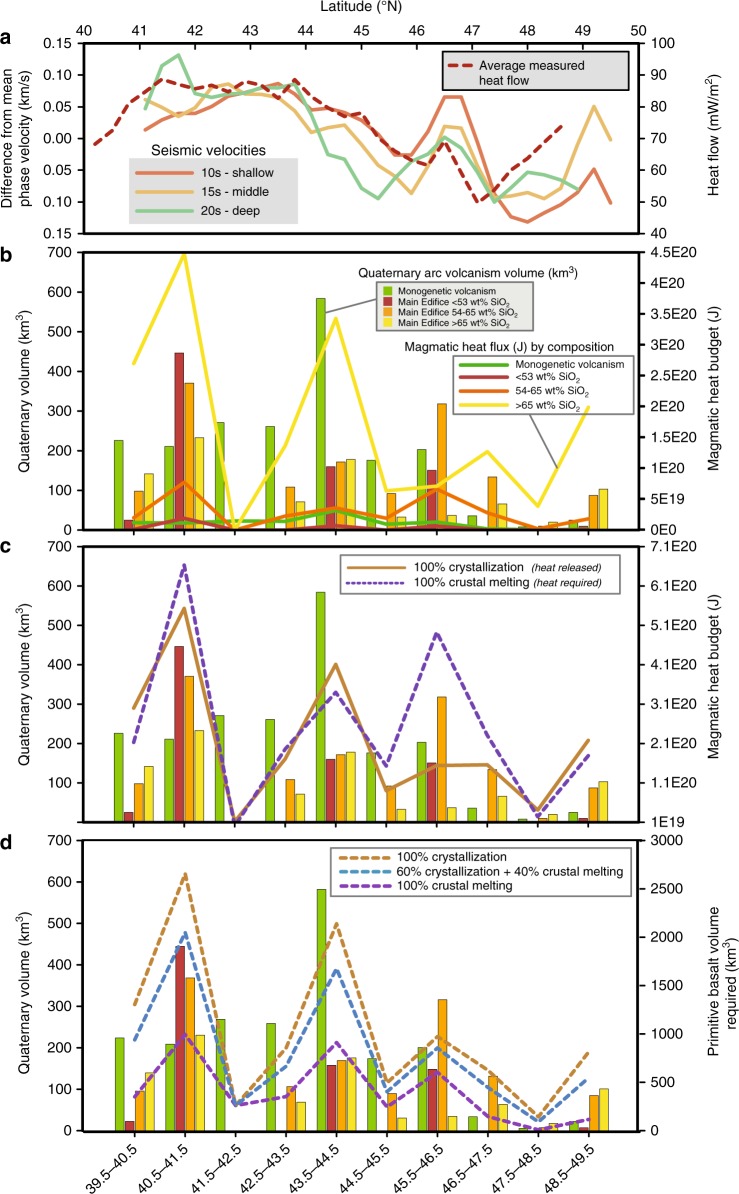
Results of our heat and flux calculations. **a** Earthquake and ambient noise seismic phase velocities from Janiszewski^51^ and measured heat flow from Ingebritsen and Mariner^86^. **b** Histogram of the quantity and composition of Quaternary arc volcanism plotted in 1° latitude bins (i.e., samples in 40.5–41.5°N plotted in the associated histogram bin) and the calculated magmatic heat budget (curves) required to generate each compositional category by pure fractional crystallization following the methods described in text. Note the dominant role of silicic magma generation in the overall heat budget. **c** Calculated magmatic heat budget (curves) required to generate Quaternary Cascades volcanism (histogram) by 100% fractional crystallization vs. 100% crustal melting. **d** Volume of mantle basalt (curves) required to generate Quaternary Cascades volcanism (histogram) via varying amounts of crystallization and crustal melting

In the Cascades, there are two predominant styles of magmatism: larger volcanic edifices, which are dominated by intermediate and silicic magmas, and dispersed monogenetic volcanic fields, dominated by mafic magmas^14,15^ (Fig. 1). Erupted volumes in both these classes vary substantially along strike, with both showing significantly greater volumes in the southern portion of the arc. To quantify these variations, we utilized a dataset of over 11,800 published major-element chemical analyses for Quaternary volcanics to characterize the average composition (<53 wt% SiO_2_, 53–65 wt% SiO_2_, >65 wt% SiO_2_ at the main arc edifices, and <53 wt% SiO_2_ monogenetic volcanism) within a series of 1° latitude bins along strike in the Cascades (Fig. 4b). To avoid the problem of greater weighting by oversampled regions, such as Mount St. Helens, we used a Monte Carlo analysis with weighted bootstrap resampling^42^ to develop a resampled dataset of Cascades volcanic rock compositions with approximately equal latitudinal sampling (see the Methods section and Supplementary Information). We use this resampled data to estimate the heat budget associated with Quaternary volcanism along the arc by two end-member scenarios: differentiation and crustal melting. In the differentiation scenario, mantle-derived basalts stall in the crust and release heat as they crystallize to produce the magmas ultimately erupted, such that the total basalt flux, the storage depths, and the extent of crystallization dictate the crustal thermal structure. In the crustal melting scenario, heat released from mantle-derived basalts causes lower crustal melting to produce the erupted magmas, such that the basalt flux, melting locations, and the extent of melting dictate the crustal thermal structure. For the differentiation calculations, experimental liquid lines of descent for a range of primitive Cascades magmas (from Medicine Lake, Mt. Shasta, Newberry, and Mt. Rainier) determined over a range of intensive variables (i.e., H_2_O content, *f*O_2_, and pressure) were parameterized to calculate the magmatic temperature and crystal content for the observed distribution of erupted volcanics^43–46^. These quantities are then used to calculate the heat input (J/kg of magma) into the crust contributed by sensible heat (i.e., heat loss due to the total temperature change) and latent heat (heat produced during crystallization) for the observed Quaternary volcanic samples. The heat calculations are then scaled according to the Quaternary volumes for these compositional categories and used to calculate the net crustal heat input resulting from Quaternary volcanism along strike (see Methods). For these calculations, we utilized the Quaternary volcanic volumes from Hildreth^15^. As discussed in the section “Future directions” below, estimation of volcanic volumes is one area where considerable refinement of the existing data sets could occur, although we note that similar calculations using volumes derived from Sherrod and Smith^14^ produce broadly comparable results (Supplementary Figures S1 and S2). To calculate the heat required to generate the observed erupted volcanic compositions via pure crustal melting rather than crystallization (although unrealistic, this represents one end-member set of calculations), the amphibolite melting experiments of Wolfe and Wyllie^47^ were parameterized to produce a relation between SiO_2_ contents, melt fraction, and temperature (see Methods). Crustal melting was assumed to be negligible for magmas with SiO_2_ less than 53 wt%. Detailed petrologic studies of silicic volcanism at Medicine Lake, Mt. Shasta, and Mt. Hood volcanoes suggest that they are produced via 0–50% crustal melting^16,32,48–50^.

Because of the comparatively large sensible heat and latent heat contribution to the crust necessary to produce silicic magma compositions (>65 wt% SiO_2_) via crystallization, the generation of silicic volcanism is the dominant signal in our calculations of the Quaternary magmatic heat budget (Fig. 4b). Because the relatively large estimated volume of monogenetic volcanism in the southern Cascades is predominantly mafic, its contribution to the crustal heat budget remains smaller than that of the erupted silicic magmas. The latent heat released during crystallization is also likely to drive crustal melting, and thus any contribution of crustal melting to the generation of the erupted compositions will lower the net calculated magmatic heat budget (see Methods). However, for any scenario with coequal to greater crystallization relative to crustal melting, the Quaternary magmatic heat budget south of 45°N is more than twice that to the north (Fig. 4c). A Monte Carlo approach suggests uncertainties in the magmatic heat calculations of 10–50% depending on the compositional category and differentiation process, which although large are much less than the factor of two along-strike variation (see Supplementary Information).

One advantage of our approach is that we can quantitatively compare geophysical data with erupted volumes and geochemical estimates of heat flux along the arc (see Methods). Significant correlations (*P* < 0.05) exist between seismic velocities and average surface heat flow, and between seismic wave speeds, erupted volumes, and the estimated thermal effects of magmatism, and many other parameters correlate with slightly lower levels of significance (*P* < 0.20). The recent deployments of the EarthScope Transportable Array, offshore Cascadia Initiative ocean-bottom seismometers and other arrays have enabled comprehensive high-resolution surface wave imaging of the crust and the upper mantle structure throughout the region^51^ (see Methods). Measured heat flow corresponds with slow seismic wave speeds from ambient noise (10–15-s periods) and earthquake (20–60-s period) surface-wave phase velocity maps, which in turn correspond with the regions of higher silicic magma production in the arc south of 45°N. Importantly, the longer (15–60 s) Rayleigh-wave phase velocities, which have peak sensitivity to wavespeeds in the middle to lower the crust and mantle, show the strongest correlation with measured heat flow, calculated magmatic heat, and erupted volumes. Calculated magmatic heat budgets correlate most strongly with seismic wave speeds at 32–60 s. Such correlations suggest that magma storage in the upper mantle and/or deep crust plays a critical role in generating the observed crustal seismic structure and heat flow (Fig. 4a, d), and argue against an important role for the upper and middle crust. This finding is broadly consistent with recent modeling by Rees Jones et al.^41^ that argues for advective heat transport by magma as the primary control on crustal heat flow in arc settings, and suggests that such advective heat transport may be the primary control on seismic wave speeds.

**Fig. 5:**
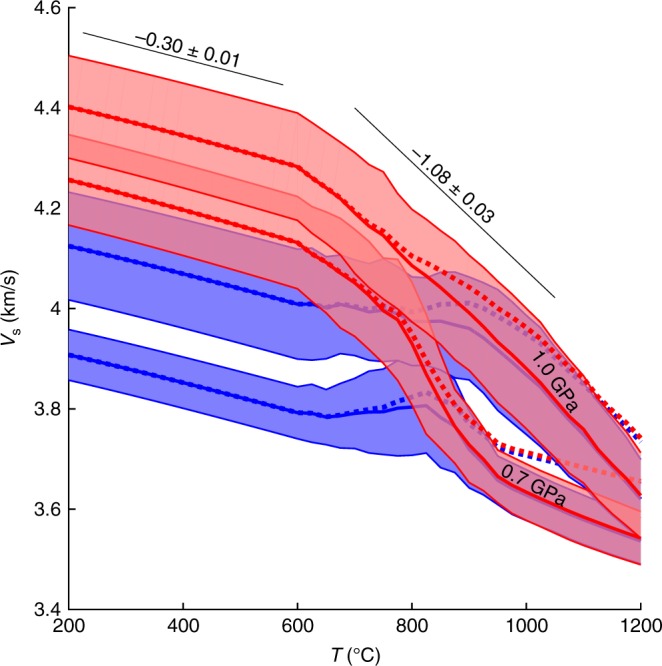
Calibration of *V*_s_—temperature relationship for typical Cascadia basement rocks. Velocities are calculated for a suite of 16 reported major-element compositions for the Siletz-Crescent basaltic terranes^86,87^, which are likely basement for the Cascadia forearc^88^. Modal mineralogy is calculated for each sample’s major element composition via the free-energy minimization code Perple_X^89^ at a range of pressures and temperatures, and parameters such as *V*_s_ and density are calculated^90^. Because chemical equilibrium is unlikely at low temperatures, below 600 °C the modal compositions are fixed to those at 600 °C (500 °C for when H_2_O is present). At high temperatures additional anelastic velocity reduction is incorporated by scaling gabbro to olivine anelasticity through their creep properties (see Methods). Red: water-free calculations; blue: calculations with 2 wt% H_2_O; thickness of swath includes one standard deviation of variation among the 16 samples. Dotted lines show results neglecting physical dispersion. Calculations shown at 0.7 and 1.0 GPa as labeled. Note change in slope, labeled, between −0.30 ± 0.01 m/s/K at low temperatures where only mineral elasticity effects velocities, to −1.08 ± 0.03 m/s/K for dry conditions at temperatures where petrologic and anelastic effects also contribute. In modeled compositions with 2 wt% H_2_O the effect is more complicated and the overall trend is midway between these extremes

We can further explore the link between the geophysical and geochemical datasets quantitatively through a 1D steady-state thermal model. At periods of 10–60 s a band of low-phase velocities closely follow the volcanic arc, a feature that is best understood as a signature of the heat input to the arc—it is otherwise difficult to explain lithologically. When averaged in a 140 km wide corridor around the modern arc, phase velocities systematically increase from south to north at all periods by 0.15–0.2 km/s, a variation that strongly correlates with heat flow and inferred magmatic heat input (Fig. 4a). The fundamental-mode Rayleigh-wave phase velocities can be quantitatively converted to heat flux given an assumed relationship between shear wave velocity (*V*_s_) and temperature (*T*), a model of *T* increases with depth, and an appropriate sensitivity kernel relating depth variations in *V*_s_ to phase velocity at a given frequency (see Methods). These calculations suggest a ~25–35 mW/m^2^ variation in heat flow, or a ~300–400 °C variation in temperature at the Moho along strike, decreasing to the north, consistent with heat flow observations^18^. These values consider both equilibrium-petrologic and anelastic reductions to *V*_s_ with increasing *T* (see Supplementary Materials), or a velocity–temperature derivative d*V*_s_/d*T* ~0.6–0.8 m/s/K for typical crustal rocks (gabbros) (Fig. 5).

The comparison of heat calculated from the observed volcanic record and via our thermal model can also provide insight into the nature of the crustal magma plumbing systems. When integrated across strike by 25–50 km and in time over the Quaternary (2.6 Ma), the seismic velocities suggest magmatic heat input into the crust of 6 × 10^21^–12 × 10^21^ J for each 100 km along strike in the arc. The fractional along strike variation in this seismic estimate of heat flux is a similar magnitude to the variation in heat flux that we calculate from the volcanic record (1 × 10^19^–7 × 10^20^ J per ~100 km: Fig. 4c). However, the absolute seismic heat estimates and those from heat flow are an order of magnitude larger than the magmatic flux estimates. This difference can be accounted for by the intrusive magmatic input never erupted (and thus not included in our analysis based on the observed volcanic record) along strike, which are typically with 3–30 times the quantity of the extrusive magmas^52^ and dominantly silicic in composition^53^. The similar fractional variation in the heat flux predicted by the seismic velocities along strike also implies that the intrusive:extrusive ratio varies by less than a factor of two along strike in the Cascades Arc.

Finally, the erupted compositions and volumes of Quaternary Cascades Arc volcanism can also be converted into the required flux of mantle-derived basalt along strike, which varies between ~60 and 1900 km^3^/myr per degree latitude (or approx. <1–10 km^3^/km/myr). Calculated this way, the mantle magmatic flux south of 45°N is at least twice that to the north (Fig. 4d), and also correlates with seismic velocities (see Methods). Our estimates of mantle flux into the Cascades crust are also approximately one order of magnitude lower than geologic estimates^52,54–56^ and constraints from numeric models^39,41,57^. Again, the most likely explanation for this is that our calculations do not include the mantle flux required to produce an intrusive complement to the observed Cascades Quaternary extrusive record. Alternatively, if we assume that the volume of the growth of the modern edifices in the Cascades dominantly reflects growth in the last 600,000 years due to preservation issues, the flux estimates then overlap with the flux constraints from other methods.

## Who drives the bus: crust or mantle?

Returning to the initial question of the relative role of crustal vs. mantle processes in driving arc diversity, the results of our case study suggest the twofold along-strike variability in the volume and heat input of mantle-derived magmas into the Cascades crust regulates the observed volcanic activity, even for intermediate and silicic magmas. This finding is similar to that from the recent numerical modeling effort of Karakas et al.^39^, which suggests that the magmatic duration and intrusion rate are the dominant variables controlling the development of upper crustal magma reservoirs capable of producing silicic magmas. The mantle melting processes beneath arcs—flux and decompression melting of the mantle ± melting of the slab—exert a first order control on the melt productivity, melt major and trace element composition, magmatic H_2_O content, and melt transport mechanism (e.g., see reviews^11,58^). Thus, along strike variations in incoming plate hydration, mantle flow patterns, thermal structure, and composition are likely to cause variations in the flux of mantle basalts into the crust, and to then strongly effect crustal magmatism and volcanism. This hypothesis can be tested with future numerical models of mantle melting in arc environments based on observational constraints on incoming-plate structure^59^, and through comparison to other arcs where the mantle input is well expressed.

Crustal structure and stress regime have also been demonstrated to play a role in modulating the mantle-derived magmatic flux and their compositional evolution in arcs^8–10,16,60,61^. The coexistence of the higher calculated magmatic heat budget, higher measured heat flow, and larger negative seismic anomalies south of 45°N (Fig. 4) suggests a larger net volume of crustal magmatic storage in a region where the arc is extensional–transtensional, such that crustal stresses do not appear to be the primary control on the intra-arc volcanic behavior. This hypothesis can be further tested by examining patterns of upper and lower crustal magma storage along strike and comparing these patterns to crustal density and thermal profiles (derived from seismic velocities and geologic constraints) to determine if magma buoyancy vs. crustal stress state dictate the patterns of magma storage.

The results of our calculations also support a hypothesis that the crustal heating from advective heat transport and magmatic differentiation is the predominant signal evident in the geophysical observations of arc crust. The fact that erupted volcanic volumes and the calculated heat budget related to observed volcanism along strike are statistically correlated with seismic velocities suggests that they may have a common cause, a finding that is supported by both the similarity in the absolute values of the heat flux calculations from seismic velocities and the volcanic record (Fig. 4a, c), and by from recent modeling studies^41^. This finding, as well as the inferred important role of the mantle in controlling arc volcanic diversity, can be further tested by probing other arcs with substantive geophysical and geochemical datasets, as well as by additional numerical studies. Preliminary investigations of geophysical studies with similar period ranges to the data used herein suggest that the Andean and Japanese arcs reveal similar (Japan) to much larger (Andes) variations in surface wave speeds along strike as the Cascades Arc^36,62–64^.

## Future directions

Over the last 50+ years, the fields of petrology and geochemistry have produced dramatic leaps in our understanding of the way that magmas are produced and evolve within subduction zones. However, we believe that the time has also come to prioritize developing a greater understanding of processes that produce arc-scale diversity in the volcanic and magmatic records of subduction, even as we continue to develop our knowledge of the behavior of individual arc volcanic systems. With the increasing availability of high-quality regional geophysical and geochemical datasets, the time is ripe for such a shift and there are a variety of evolving research strategies that could be employed to do this. The approach taken here—identifying common parameters that can be used to quantitatively compare regional scale geochemical and geophysical datasets—is one such promising strategy.

Long standing questions regarding the controls on the positioning and the size of individual volcanic edifices along an arc, the lifetime of individual arc volcanoes, and the causes of different volcano “personalities”^34,65^ could also benefit from this approach. As in our case study, variations in mantle flux vs. upper plate control are potential competing drivers of volcano location, migration, size, and evolutionary patterns over time. Although our results suggest that deep crustal and mantle processes exert the dominant controls on Cascades volcanic diversity, the characteristics of individual volcanoes and volcanic systems are still likely to be strongly modulated by crustal parameters such as stress state, rheology and density profile^16,66–68^ and by the position of magma storage zones within the crust^10,34^. Thus, one useful approach would be to utilize crustal density and magma buoyancy as common intensive variables to quantitatively compare thermobarometric constraints from erupted magmas to regional models of crustal density structure from geophysical observations. In this case, numerical modeling would also be an important component. This would involve dynamic models of crustal magma emplacement and storage^68^ that utilize observed crustal density structure as an initial condition, and where the location of magma emplacement is dictated by relative buoyancy and rheology.

In addition to the multidisciplinary approach laid out here, our study illustrates the need to prioritize addressing key areas of uncertainty within specific disciplines. For example, geoscientists must tackle problems of sampling bias and ergodicity (the ability to show that the available sample is representative of the larger data set). Geochemical datasets are often highly heterogeneous in sampling density, being governed by the locations of volcanoes, timing of eruptions and availability of suitable samples and sample locations, as well as general interest of selected locations. One advantage of the arc-wide perspective that we have taken is that it allows us to compare the observed volcanic record to regional-scale geophysical data sets. However, this approach is also complicated by the nonrepresentative sampling involved in most geochemical data sets. While large number of geochemical data are available for arc rocks^69^, exceedingly few sampling programs are designed to provide representative coverage, and tend to focus on volcanoes or rock units of specific interest. Application of weighted bootstrap and other techniques^42,70^ can be used to minimize the effect of this sampling bias, however, strategic resampling of less well-known areas, and selection of sample localities on the basis of geochemical and spatiotemporal representativeness can also help address this source of uncertainty.

Another critical data set is the volumes of erupted magmas within individual arcs. In our study of the Cascades we focused on volcanic volumes, but plutonic volumes are also relevant in more dissected arcs. Volumes allow us to convert from observed compositions to measures representative of the productivity and output of an arc over time, but are also one of the most difficult quantities to constrain. For our study, the most critical datasets were the volumetric estimates of Quaternary arc volcanism along strike within the Cascades, particularly for the fields of multiple small monogenetic centers. Despite this being one of the best characterized arcs in the world, there are significant discrepancies between the two existing volumetric datasets^14,15^, which mainly revolve around questions of the quantity of distributed monogenetic volcanism and glacial erosion along strike. The combination of recent and future regional studies on the rates of erosion in the Cascades and the opportunity to utilize LIDAR and other high-resolution satellite imagery to estimate volumes of monogenetic volcanism suggests there is opportunity to improve volumetric estimates, and apply similar approaches to other arc systems.

From the seismic imaging perspective, joint inversions are necessary to resolve the inherent depth smoothing of surface waves and improve the depth resolution of velocity anomalies, and may require greater density of seismometers near volcanic areas. Surface waves are subject to tradeoffs between Moho depth and velocities, such that Moho variations could be misinterpreted as temperature variations. Joint inversions with signals such as receiver functions provide help in resolving these ambiguities—for example fully nonlinear Bayesian inversions allow data to drive complexity in structure^71,72^. Such inversions should target higher resolution constraints on *V*_s_ within the lower crust, which would better assess the intrusive:extrusive ratio and the crustal density structure. Lateral resolution is also an issue as volcanic systems may be dominated by structures of much smaller scale than the several tens of km typical of seismic arrays—addressing this problem requires much denser arrays of seismographs, such as “large-N” arrays that take advantage of easily portable instruments available in large number^73^. Finally, while our ability to interpret seismic data is improving in dry systems (e.g., Fig. 5) the effects of melt on seismic waves remains ambiguous, particularly for silicic magmas. Careful integration of other observations, such as magnetotelluric data, has potential to resolve some of these ambiguities^74^.

In closing, we suggest that, in addition to ongoing focused studies of individual volcanic systems, important new opportunities are emerging to use petrological, geochemical and other data sets to address outstanding problems related to arc volcanism in aggregate form. These include investigations of the underlying causes of regional and arc-scale variations in volcanism and magmatic phenomena in time and space. Doing so effectively requires greater integration between geophysical, geochemical and petrological data sets, as well as important contributions from other disciplines such as numerical modeling. The varying but complementary spatial and temporal sensitivities of these disciplines, and quantitative relationships between them, will help us break the “Emoji” vs. “Disembodied” volcano paradigms, and thus represents tremendous potential to explore new frontier questions and to advance subduction zone science.

## Methods

### Cascades volcanic geochemistry data set

We used the Quaternary Cascades volcanic rock compilation of Pitcher and Kent^76^, taken primarily from Earthref.org, which includes samples from USGS and literature sources. Samples were filtered for whole rock analyses (excluding glasses) with SiO_2_ and at least six other major elements reported. We used Chauvenet’s criterion for each major element to remove outlier analyses and also removed a very small number of samples that were clear outliers (largely related to obvious transcription errors) from visual inspection of bivariate plots of SiO_2_ vs. other major elements, resulting in a dataset of 11,866 analyses of volcanic rock. Monte Carlo analysis with weighted bootstrap resampling^42^ was used correct for the unequal distribution of samples along strike (Supplemental Fig. 1). Note that this procedure only corrected for distributions in latitude and not in time, as the set of samples with reliable ages is too small to provide adequate age resolution within the Quaternary. For our resampling procedure, we followed that of Pitcher and Kent^76^ where samples were divided into latitudinal bins of 0.25° width between 40.0–50.5° latitude (Figure S3). Samples from each bin were assigned a weight *w*_*i*_ inversely proportional to the number of samples in that bin,

$$w_{i = }\frac{1}{{n_{\mathrm{bin}\;i}}}n_{\mathrm{min}}{,}$$

where *n*_bin *i*_ is the number of samples in latitude bin *i*, and *n*_min_ is the number of samples in the smallest bin. The weight for the smallest bin is equal to 1. To avoid overweighting samples from bins with small *n*_bin *i*_ we did not include data from three bins with *n*_bin *i*_ of 9, 3, and 5 total samples (latitude bins 40.0–40.25, 50.25–50.5, and 49.0–49.25) were not included in the bootstrap procedure. The smallest bin size used was *n*_bin *i*_ = 22 for latitude bin 40.75–50.0°. In addition, no data was available for bins 49.25–49.50° and 49.50–49.75°. To produce our synthetic data set, 50,000 samples were then randomly selected with replacement from our compiled data set, such that the probability of inclusion of samples from each latitude bin was directly proportional to calculated weight for that bin. This resulted in greater sampling from bins with fewer samples available. The whole arc average compositions of three compositional bins (<53 wt% SiO_2_, 53–65 wt% SiO_2_, and >65 wt% SiO_2_) were determined from this synthetic data set. The posterior distribution produced by this approach is compared to the observed sample numbers per bin in Supplementary Figure S3.

### Uncertainties in heat calculations

We assessed the uncertainty in the calculated heat related to crustal melting and fractional crystallization using a Monte Carlo approach. For each of the three compositional bins (<53% SiO_2_, 53–65 wt% SiO_2_ and >65 wt% SiO_2_) used for fractional crystallization and melting calculations we repeated the melting and fractional crystallization calculations 10,000 times varying the input SiO_2_ or FeO*/MgO content within a normal distribution defined by the average and standard deviation calculated for that compositional bin. We then calculated standard deviations for calculated heat. To test sensitivities to other parameters for crustal melting we also performed an additional set of calculations where we randomly varied the solidus and ambient crustal temperatures around the selected values of 800 and 600 °C using a given standard deviation. We did not assess variation in the values of heat capacity (1100 J kg^−1^ K^−1^) and enthalpy of fusion (300,000 J K^−^^1^), treating these as constants. One limitation to the Monte Carlo approach is that the frequency distribution of SiO_2_ contents with each compositional bin do not follow strictly Gaussian distributions. The basaltic (<53 wt% SiO_2_) and silicic (>65 wt% SiO_2_) compositional bins approximate Gaussian distributions, but the intermediate composition bin (53 <wt% SiO_2_ <65) is skewed to lower SiO_2_ concentrations (Figure S1). To test the effect of this we also evaluated uncertainties by varying input SiO_2_ contents randomly within the interval defined by mean average deviation (MAD; a more robust measure of variability for non-Gaussian distributions) for SiO_2_ in each compositional bin. The resulting magmatic heat estimates typically exhibited normal or close to normal distributions, but we assess both standard deviations and MAD values below. The largest source of uncertainty is the range of SiO_2_ and FeO*/MgO values shown by samples in each compositional bin, and the sensitivity of calculated heat values to changes in SiO_2_ and FeO*/MgO also varies between the different compositional bins used.

For the crustal melting calculations, using the observed standard deviations and MAD for the SiO_2_ contents of samples in the silicic compositional bins equates to standard deviations in calculated heat produced of 39% and 33%, respectively. For the intermediate compositional bin the variation in calculated heat values corresponds to standard deviations and MAD values heat of 8 and 7%. The effect of variations in the estimated solidus and ambient crustal temperatures to the overall uncertainties in heat values calculated from crustal melting is relatively small. Using a relatively large standard deviation of 50 °C for both the solidus and ambient temperatures (were we use values of 800 and 600 °C for these, respectively) adds only an additional 3–5% uncertainty. If we use uncertainties of a more realistic ±25 °C in these temperatures it adds only 1–2% additional uncertainty.

For the fractional crystallization calculations, using the observed standard deviations and MAD for the SiO_2_ contents of samples in the silicic compositional bin gives standard deviations and MAD values in calculated heat contents of 15% and 11%, respectively. For the basaltic compositional bin the uncertainties in heat values calculated for dry crystallization using FeO*/MgO ratios are relatively high, with standard deviations and MAD of 55 and 48%, although the corresponding absolute heat values are lower. This is the result of the exponential dependence of temperature on the liquid FeO*/MgO for experiments documenting the tholeiitic liquid of descent. For the intermediate compositional bin the standard deviations and MAD values for calculated heat contents are 56% and 47%, respectively.

### Crystallization calculations

Experimental liquid lines of descent for primitive Cascades magmas from Medicine Lake, Mt. Shasta, Newberry and Mt. Rainier were parameterized to track magmatic temperature and crystal content as a function of liquid composition (Figure S4). The parameterization for nominally anhydrous differentiation is based on the experiments of Grove et al.^43^, the hydrous parameterization on the experiments of Grove et al.^44^, and two damp or intermediate water content parameterizations based on the experiments of Blatter et al.^45^ and Mandler et al.^46^. The independent variable for the hydrous parameterization is SiO_2_ rather than FeO*/MgO because of the stronger correlation between hydrous liquid composition and SiO_2_ in the calc-alkaline differentiation trend.

Crystallization of a primitive arc basalt with >4.5 wt% H_2_O at 2 kbar^44^

1 $${T} = - 36.15\,\left( {\mathrm{SiO}_{2}} \right) + 3285.7\,\,\,\,\,\,R^2 = 0.77{.}$$

2 $$\% {\mathrm{Xls}} = - 0.4375\left( {T} \right) + 475.08\,\,\,\,\,\,\,\,R^2 = 0.87{.}$$

Crystallization of a primitive arc basalt with 2 wt% H_2_O at 9 kbar^45^

3 $${T} = - 121.37\left( {{\mathrm{FeO/MgO}}} \right) + 1303.6\,\,\,\,\,\,\,{R}^2 = 0.97{.}$$

4 $${\mathrm{\% Xls}} = - 0.4204\left( {T} \right) + 495.17\,\,\,\,\,{R}^2 = 0.95{.}$$

Crystallization of an arc magma with 6 wt% H_2_O at 2 kbar^46^

5 $${T} = - 96.41\left( {{\mathrm{FeO/MgO}}} \right) + 1300\,\,\,\,\,\,\left( {2\,{\mathrm{data}}\,{\mathrm{points}},{R}^2\,{\mathrm{not}}\,{\mathrm{meaningful}}} \right){.}$$

6 $${\mathrm{\% Xls}} = - 0.318\left( {T} \right) + 318.18\,\,\,\,\,\,\left( {2\,{\mathrm{data}}\,{\mathrm{points}},{R}^2\,{\mathrm{not}}\,{\mathrm{meaningful}}} \right){.}$$

Crystallization of a nominally anhydrous primitive basalt^43^

7 $${T} = - 98.51{\mathrm{ ln}}\left( {{\mathrm{FeO/MgO}}} \right) + 1218.5\,\,\,\,\,\,{R}^2 = 0.77{.}$$

8 $${\mathrm{\% Xls}} = - 0.582\left( {T} \right) + 697.6\,\,\,\,\,\,{R}^2 = 0.99{.}$$

where (SiO_2_) is in wt%, (FeO/MgO) is calculated using FeO* in wt% and (*T*) is temperature is in °C. Given the similarity of the wet vs. dry liquid lines of descent for these samples, for simplicity we use the anhydrous parameterization of Grove et al.^43^ for the mafic (<53 wt% SiO_2_) and intermediate (53–65 wt% SiO_2_) samples in the calculations presented in main text Fig. 4. Experiments and studies of arc volcanism in the central to southern Cascades have shown that in order to reach the high SiO_2_ contents of rhyodacites to rhyolites, wet parental magmas are required^49^, thus the hydrous Grove et al.^44^ parameterization was used to calculate the liquid line of descent for the silicic samples (>65 wt% SiO_2_).

### Crustal melting calculations

To estimate temperature and extent of crustal melting necessary to produce the observed erupted compositions, the experiments Wolf and Wyllie^47^ for a Mg-rich amphibolite bulk composition at 10 kbar were parameterized to produce a relation between SiO_2_ content, melt fraction and temperature. Use of amphibole and experiments at this pressure assumes that melting occurs in the lower crust^9^. The average *T*_melt_ and *f*_melt_ were calculated from the bootstrap average SiO_2_ values for the compositional ranges of 53–65 wt% SiO_2_, and >65 wt% SiO_2._ We assumed that compositions <53 wt% SiO_2_ were primary magmas or produced solely from fractional crystallization):

9 $${T}_{{\mathrm{melt}}} = - 0.5945\,\left( {{\mathrm{SiO}}_2} \right)^2 + 61.054\,\left( {{\mathrm{SiO}}_2} \right) - 565.1\,({R}^2 = 0.81){.}$$

10 $${f}_{{\mathrm{melt}}} = - 0.0012\left( {{\mathrm{SiO}}_2} \right)^2 + 0.1148\,\left( {{\mathrm{SiO}}_2} \right) - 2.1525\,\,\,\,\,({R}^2 = 0.76){.}$$

### Magmatic heat calculations

The results of the crystallization parameterizations were used as the input values to calculate the heat released (J/kg of magma) into the crust from both the sensible heat (i.e., heat loss due to total temperature change) and latent heat (i.e., heat produced during crystallization), following Sparks and Marshall^77^ (Eq. (11)) (Figure S5). Likewise, the results of the crustal melting parameterizations were used as input to calculate the heat required to melt lower crustal amphibolite following Grunder^54^ (Eq. (12)).

11 $${\mathrm{For}}\,{\mathrm{crystallization}}:{\mathrm{Heat}}\left( {{\mathrm{J/kg}}} \right) = {C}_{\mathrm{p}}\left( {{T}_{{\mathrm{start}}} - {T}_{{\mathrm{final}}}} \right) + \left( {{f}_{{\mathrm{crystal}}}} \right){H}_{\mathrm{f}}{.}$$

12 $$ {{\mathrm{For}}\;{\mathrm{crustal}}\,{\mathrm{melting}}:{\mathrm{Heat}}\left( {{\mathrm{J/kg}}} \right) = {C}_{\mathrm{p}}\left( {{T}_{{\mathrm{melt}}} - {T}_{{\mathrm{solidus}}}} \right) + {H}_{\mathrm{f}} \, \bullet \, {f}_{{\mathrm{melt}}} + {C}_{\mathrm{p}}\left( {{T}_{{\mathrm{solidus}}} - {T}_{{\mathrm{crust}}}} \right).}$$

Where *C*_p_ is the heat capacity (1100 J/(kg K)) and *H*_f_, the heat of fusion (3 × 10^5^ J/kg) following Grunder^54^. For crystallization, *T*_start_ is the initial temperature of the fractionating magma (here we use 1200 °C), *T*_final_ is the temperature calculated from the FeO*/MgO ratio or SiO_2_ content above, and *f*_crystal_ is the calculated crystal fraction. For melting, the solidus temperature (*T*_solidus_) is 800 °C and the ambient crustal temperature (*T*_crust_) is 600 °C (equivalent to 20–30 km depth at a geothermal gradient of 20–30 °C/km). We also assume that the heat capacities of the solid and liquid phases are equal^77^. *T*_melt_ is the calculated temperature of melting determined using Eq. (9) and *f*_melt_ the melt fraction calculated using Eq. (10). Uncertainties in calculated heat contents and the equivalent volume of basalt required were assessed using a Monte Carlo approach as discussed above.

The total Quaternary heat budget along strike associated with Cascades volcanism was determined by weighting the calculated sensible + latent heat contribution to the crust from crystal fractionation and crustal melting for each of the compositional categories used (<53 wt% SiO_2_, 53–65 wt% SiO_2_, and >65 wt% SiO_2_) by the total Quaternary extrusive volume for each compositional category with each latitude bin from Hildreth^15^. In order to make a semi-quantitative assessment of the relative volume of each compositional category erupted at the main edifices within a given latitude bin, the volume estimate per edifice were weighted by the relative quantities of each composition given in Hildreth^15^ Table 2 (e.g., Basalt > Andesite > Dacite). Hildreth^15^ and Sherrod and Smith^14^ both provide volume estimates for Quaternary extrusive volcanism along the entire length of the Cascades Arc and were examined for use in our calculations (Figured S1 and S2), and both sets of volume estimates produces broadly comparable results with respect to the pattern of variations in erupted volume and Quaternary heat budgets. For all latitude bins with the exception of 43.5–44.5°N, the heat released from 60% crystallization is more than necessary to produce the remainder of the erupted material through crustal melting. The volume and composition of material erupted at Newberry and Three Sisters region (latitudes 43.5–44.5°N) requires a different balance between crystallization and crustal melting, as 60% crystallization does not release enough heat to produce the remainder of magmas through crustal melting.

### Volume of mantle basalt calculations

The volume of mantle basalt required to produce the observed erupted Quaternary volcanic record through both crystallization and crustal melting was estimated using a simple back-of-the-envelope approach, where the calculated percentage of crystallization or crustal melting required to reach a given erupted magma composition was used in conjunction with erupted volumes to back estimate the total volume of mantle-derived basalt (at 1200 °C) required (Supplementary Table S1). Given the unlikelihood of >80–90% crystallization producing the erupted compositions in nature, we also calculate a more realistic scenario in which magmas are produced by a mixture of the two end member processes with 60% of crystallization and the remainder through crustal melting.

### Calculation of seismic phase velocities

Seismic phase velocities are determined using vertical-component Rayleigh wave measurements calculated from ambient noise data at the 10 and 15 s periods and from earthquake records at 20 s and longer periods. The results along the Cascades volcanic arc are constrained as part of an inversion over the broader Cascadia region. Phase velocities are measured for ambient noise from all station pairs in the frequency domain^78,79^, and estimated via Helmholtz tomography of individual earthquakes for longer periods^80,81^ (Figure S6). The phase velocities are then inverted for fundamental-mode Rayleigh phase velocity maps at a grid spacing of 0.3°, at periods from 10 to 100 s. Methods and complete results are fully described in a separate publication^51^. To examine variations in phase velocity along the Cascades Arc, we examine phase velocity maps between 120.5–122.3° W and 39–49.5°N, regridded onto a 0.2 × 0.2° grid and then averaged in 1° latitudinal bins that match the geochemical sample bins. The seismic results do not extend farther south than 40.1°N; therefore the southernmost bin only averages from 40.1–40.499°N. These are then differenced from the mean value from the entire Cascades Arc for each period; negative differences imply that the phase velocities are slower than average (Fig. 4a).

### Correlation calculations

Pearson correlation coefficients correlations and their significance were calculated between the 10, 15, 20, 32, 40, and 60 s phase seismic velocities and average heat flow data, as well as the for the monogenetic and edifice volumes and calculated magmatic heat budgets (Tables S2 and S3).

### Inferring temperature from S-wave velocities

Historically, estimates of d*V*_s_/d*T* (the change in S-wave velocity with temperature) are made at ultrasonic frequencies and are of the order −0.20 to −0.32 m/s/K for a range of igneous and high-grade metamorphic rocks, for example by comparing *V*_p_/*V*_s_ of Christensen^82^ with P-wave derivatives Christensen and Mooney^83^. However, these derivatives are likely underestimated (and temperature variation thus overestimated) through two effects. The “petrologic effect” occurs because at increasing temperatures thermodynamic equilibrium alters the modal mineralogy, often amplifying the velocity reduction with increasing *T*. Second, at high temperatures the “anelastic effect” decreases the measured *V*_s_ at increased *T* due to attenuation and the resultant physical dispersion at finite frequency^84^. To estimate both effects, we calculate (anharmonic) velocities for a suite of characteristic Cascadia forearc basement rocks at a range of temperatures, for equilibrium or near-equilibrium compositions, and then approximately correct those velocities for anelasticity (Fig. 5; Figure S7).

The petrologic effect is estimated by calculating equilibrium mineralogy for a suite of gabbroic rocks appropriate for Cascadia forearc basement, and then calculating isotropic *V*_s_ from those compositions at elevated pressure and temperature (Fig. 5, caption). The anelastic effect is estimated from well-calibrated data on olivine-rich rocks^85^ and scaling to gabbro creep rates (Figure S7). The resulting d*V*_s_/d*T* from combining both effects varies from −0.30 ± 0.01 m/s/K at low temperatures to −1.08 ± 0.03 m/s/K at high temperatures and dry conditions; wet conditions show derivatives closer to −0.5 m/s/K for wet crust. These values are close to the −0.6 to −0.8 m/s/K derived by comparing heat flow to phase velocities as described in the next section (also Figures S7 and S8) and indicate these higher d*V*_s_/d*T* are most appropriate.

### Comparing seismic velocities to heat flow

Observed average surface heat flow varies from 57 to 87 mW/m^2^ along strike (Fig. 4a; Table S2). This variation is modeled as a temperature profile which increases linearly in the crust up to some maximum mantle potential temperature (*T*_p_), such that the slope of the crustal temperature profile varies to produce the variations (see Figure S8). The observed temperature perturbations are then fit to the observed phase velocities (Fig. 4a) using the appropriate phase velocity kernels (Figure S6) and the thermal model to estimate d*V*_s_/d*T* and a geotherm. In this way, at each latitude the variation in heat flow is converted to a variation in phase velocity at each period, and a scalar d*V*_s_/d*T* is solved for along with crustal temperature. Results (Figure S8) show d*V*_s_/d*T* ranging from −0.6 ± 0.1 m/s/K at periods of 15–20 s to −0.8 ± 0.1 m/s/K at 10 s and 32 s. These *V*_s_—*T* relations are comparable to what we infer from rock physics (see previous section) and indicate an overall compatibility between seismic velocities and heat flow variations. More complex thermal models, for example including a near-surface radiogenic heat flow layer, change the absolute temperatures inferred but do not significantly change d*V*_s_/d*T* as long as the layer does not vary appreciably along strike.

These fits (Figure S8) show that that the pattern in heat flow variation can predict the seismic velocities well, explaining 65–70% of the phase velocity variance at 15–32 s periods. For models embedding a radiogenic “granite” layer in the upper 5 km, the model temperatures at 40 km depth vary from 1000–1150 °C in the southern Cascades to 650 °C in the northern Cascades (Figure S8). Varying parameters, e.g., thermal conductivity by 0.5 W/m/K and *T*_p_ by 100 °C, changes these values by <100 °C. With these caveats, the observed variation in seismic velocity corresponds to 300–400 °C variation in temperature at the Moho and roughly 30 mW/m^2^ in surface heat flow.

## Supplementary information

Supplementary Information
