# Supplementary material for: The causes of spatiotemporal variations in erupted fluxes and compositions along a volcanic arc
Source: Nat Commun. 2019 Mar 22;10:1350. doi: 10.1038/s41467-019-09113-0 (PMC6430768; doi:10.1038/s41467-019-09113-0)
Supplement: Supplementary file 1 — Supplementary Information [file 41467_2019_9113_MOESM1_ESM.pdf]

Till et al., Arc Diversity Supplementary Figures

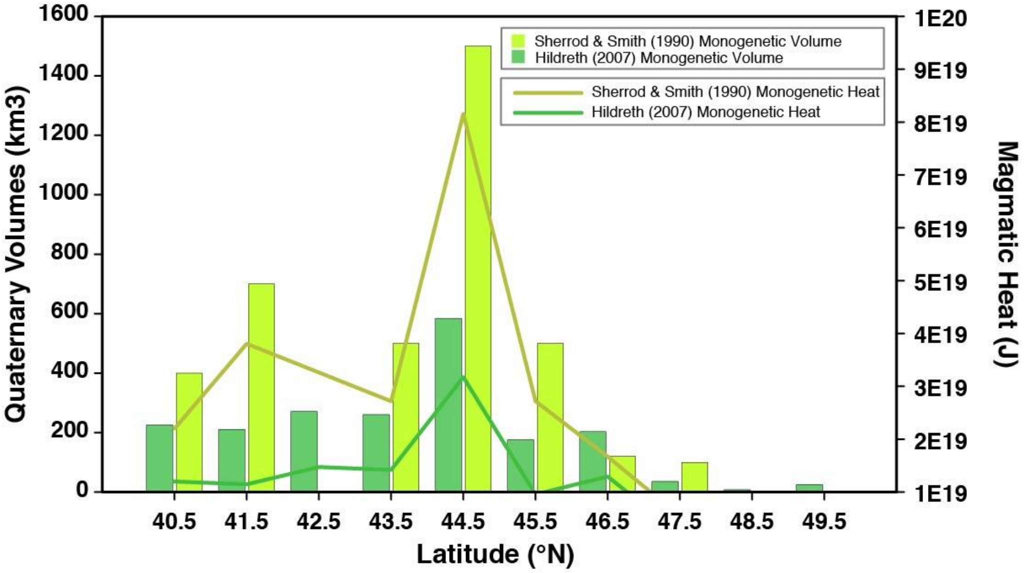

***Supplementary Figure 1**. Comparison of erupted Quaternary volumes and related heat calculations for the distributed monogenetic Quaternary volcanism in the Cascades arc, using the volumetric datasets of Hildreth[1] and Sherrod and Smith[2]. Heat calculations follow the same methods as for Figure 4 in the main text. Volcanic volumes are divided into 1-degree latitude bins, plotted at the maximum latitude of a given bin. Differences between the two datasets are in part because of differences in the quantities of monogenetic volcanism along strike, as well as in the estimates about the amount of main edifice material lost to glaciation. Hildreth[1] does not explicitly estimate the volume of distributed mafic volcanism but does include an estimate of the number of mafic vents along strike. Therefore, we use the total number of monogenetic vents for a given latitude from Hildreth[1] (Table S1) and an estimate of the average erupted volume for a given vent (~1 km^3/vent) based on case studies of several monogenetic centers (e.g., Lava Butte, OR) to approximate the total monogenetic volume. Vents associated with a rear-arc edifices where volume information was available in Hildreth[1] including Newberry and Medicine Lake volcanoes, were not included in this estimate of monogenetic volcanism as they are now known not to be exclusively mafic[3], and instead were included in the "main edifice" estimates (Table S1). Conversely, Sherrod and Smith[2] includes these rear-arc edifices in their monogenetic volcanism category — the inclusion of Medicine Lake is evident in the 40.5-41.5°N bin and Newberry volcano in the 43.5-44.5°N bin — which causes the higher crustal heat budget for monogenetic volcanism and lower crustal heat budget for main edifice volcanism for the Sherrod and Smith[2] dataset relative to that from Hildreth[1]. Although the mafic volcanism does contribute an important heat flux to the crust, the magnitude is significantly smaller per volume unit compared to the silicic volcanism as calculated using the above experimental crystallization parameterizations.*

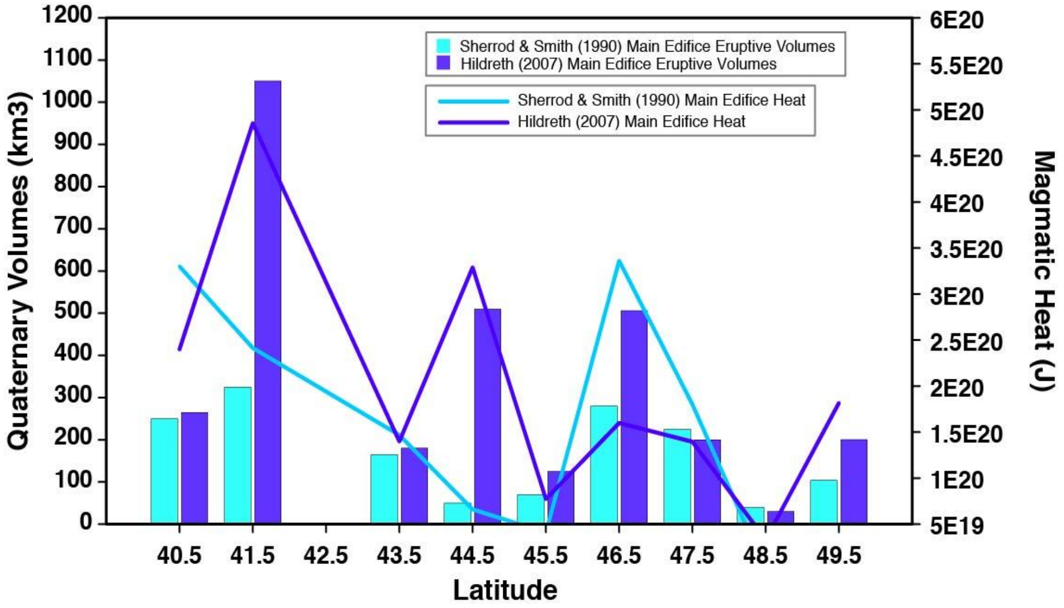

Supplementary Figure 2. *Comparison of erupted Quaternary volumes and related heat calculations for the main Quaternary volcanic edifices in the Cascades arc, using the volumetric datasets of Hildreth[1] and Sherrod and Smith[2]. Heat calculations follow the same methods as for Figure 4 in the main text. Volcanic volumes are divided into 1-degree latitude bins, plotted at the maximum latitude of a given bin. A semi-quantitative assessment of the relative volume of each compositional category erupted at the main edifices within a given latitude bin, using the relative quantities given in Hildreth[1] Table 2 (e.g., Basalt >Andesite >Dacite) and the overall volume estimates for each edifice. As Sherrod and Smith[2] only distinguishes the monogenetic mafic volcanism from that at the main edifices, and does not provide relative volumes of each composition at the main arc edifices, we use the relative quantities of each composition from given in Hildreth[1] Table 2 in conjunction with the Sherrod and Smith[2] main edifice volume estimates to produce the comparative calculations above. Overall both datasets yield similar magnitude estimates of Quaternary magmatic heat although the distributions are slightly different due to the choices and limitations of each study discussed above. Given the benefit of the additional compositional information contained in the Hildreth[1], and the overall similarity in monogenetic distributions between the two datasets with our vent to volume approximation (despite differences in absolute volume), we chose to adopt the Hildreth[1] volumes for our calculations presented in the main text. However, we also note that using either volume estimate sets produces broadly comparable results with respect to the pattern of variations in erupted volume and quaternary heat budgets.*

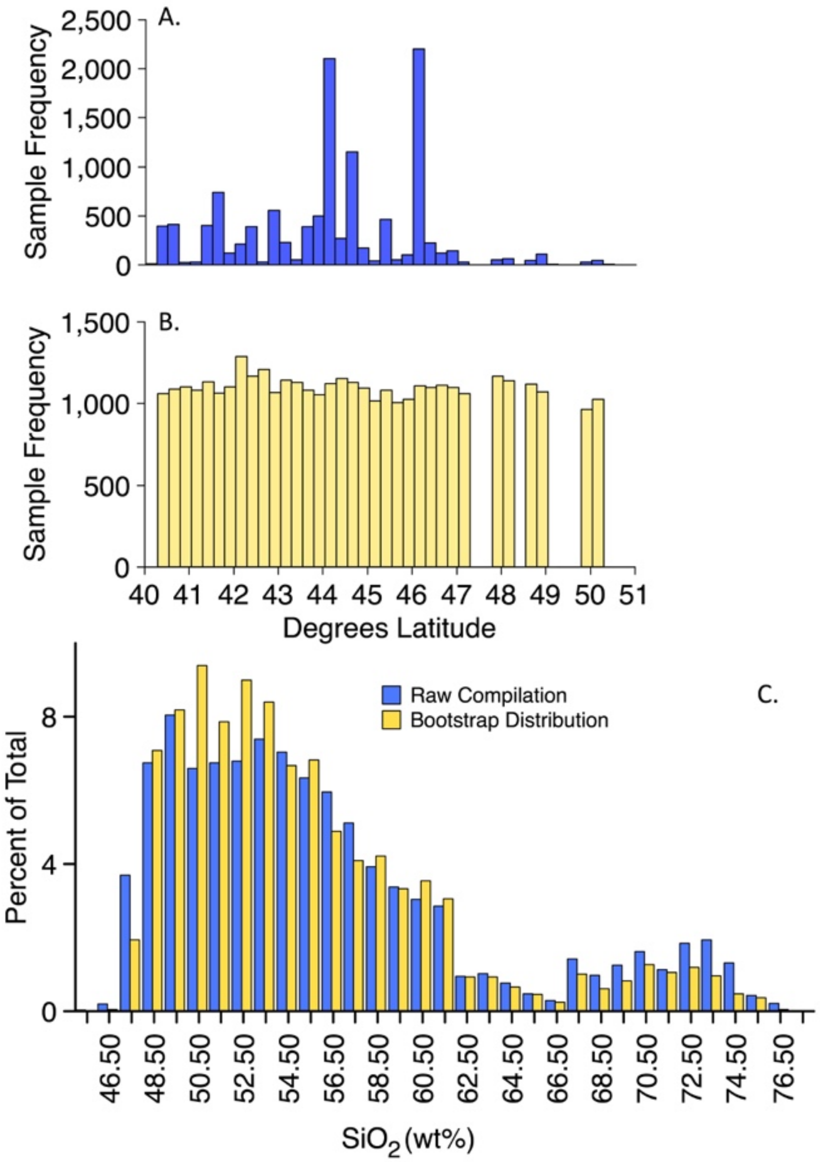

Supplementary Figure 3. *(A) Observed and (B) Posterior distribution for Quaternary Volcanic samples produced by Monte Carlo with weighted bootstrap resampling procedure. (C) Frequency histogram of* SiO_2 *contents of samples from observed and bootstrap-corrected data sets.*

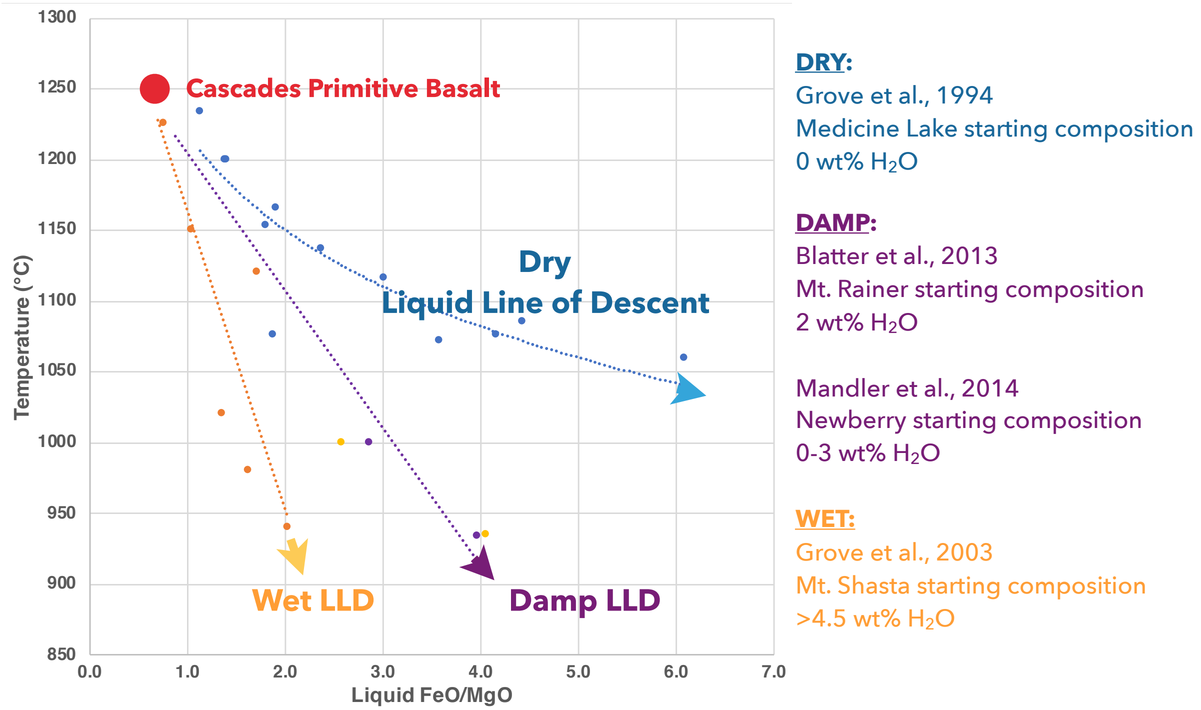

Supplementary Figure 4*. Experimentally-determined liquid lines of descent*[4-7]*. Differences in* H_2O *content of the primitive magma produce differences in the order and amount of crystallization such that liquid FeO*/MgO-temperature relationship varies. Simple linear fits were used as the focus of this study is at intermediate crystal contents (not close to the solidus or liquidus) where a linear approximation is reasonable*[8].

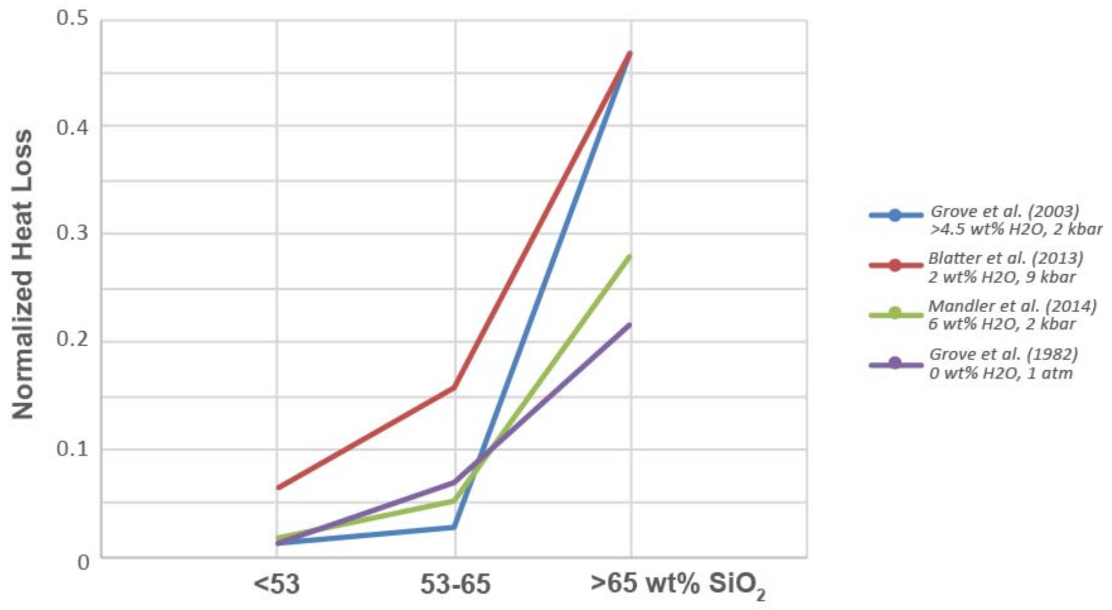

Supplementary Figure 5. *Normalized heat loss predicted by the various experimental liquid line of descent parameterizations and the bootstrapped dataset as a function of magma composition. All parameterizations, with the exception of the damp parameterization of Blatter et al.[5], predict <5% of an erupted mafic sample's initial sensible heat is lost during its journey through the crust, where the initial sensible heat is calculated as the heat capacity multiplied by the magma's temperature entering the crust in Kelvin (e.g., 1200 J/kgK * 1473 K). All parameterizations predict that intermediate (53-65 wt% SiO_2) magmas lose an average of only <10-15% of their sensible heat en route to the surface if they evolve through pure crystallization. For the nominally anhydrous liquid line of descent an arc-wide average of the heat released for the <53 wt% SiO_2 samples is ~$19{,}078^{+59{,}841}_{-19078}$ J/kg and 114,142 ± 84,561 J/kg for the 53-65 wt% SiO_2 samples. For a wet liquid line of descent, the heat released for the >65 wt% SiO_2 samples it is 770,326 ± 132,400 J/kg. Given the similarity of the wet vs. dry liquid lines of descent for these samples, for simplicity we use the anhydrous parameterization of Grove et al.[4] for the mafic (<53 wt% SiO_2) and intermediate (53-65 wt% SiO_2) samples in the calculations presented in main text Figure 4.*

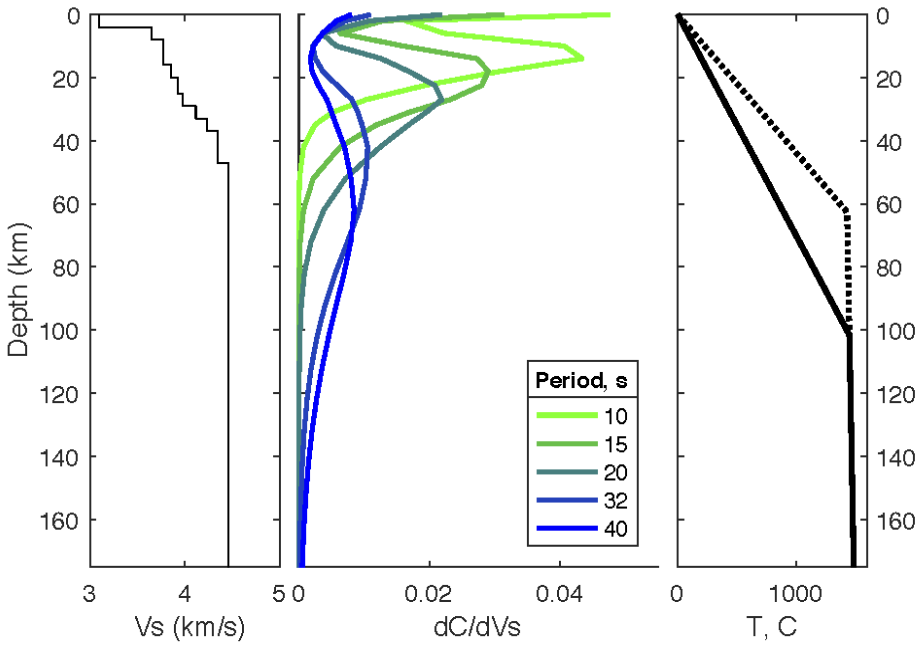

Supplementary Figure 6. *(Left) Reference velocity model for generating shear velocity (Vs) sensitivity kernels*[9-10]*. (Middle) predicted sensitivity kernels of fundamental-mode Rayleigh wave phase velocities to Vs at 10 - 40 s periods. Kernels are calculated using the program surf96*[11-12]*. These kernels are convolved with temperature perturbations (difference between solid and dashed line in right panel) to estimate a model-dependent dVs/dT. (Right) reference (solid) and perturbed (dashed) thermal model used to compare seismic velocities with heat flow. We assume that the minimum regional heat flow (q_0) of 57 mW/m^2(Figure 4a) represents the background heat flow absent magmatic addition, and that temperature T varies linearly with depth z, $T(z) = q_0z/k$, until it reaches the mantle adiabat $T_p+(0.5\ K/km)z$. Here, k is thermal conductivity and $T_p$is the mantle potential temperature. At higher heat flow the linear part is perturbed but $T_p$is not, so that $T(z) = (q_0+ \delta q)z/k$ until the adiabat is reached; δq is the difference between observed heat flow and q_0. Temperature perturbations are largest at 20-70 km depths where 15-32 s surface waves have most sensitivity. We tested a range of more complex models, including radiogenic crustal heating and more complex basal boundary conditions, and found nearly identical relationships between heat flow and dc/dT, the sensitivity of phase velocity to temperature.*

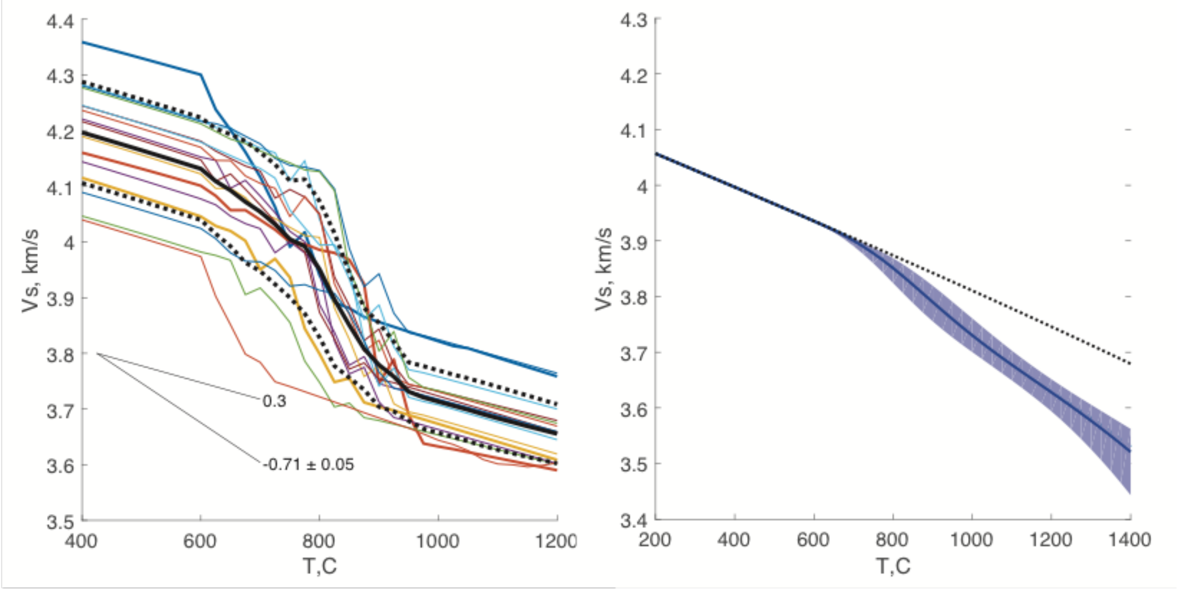

Supplementary Figure 7. *The petrologic effect and the anelastic effect, calculated at 0.7 GPa. (Left) Unrelaxed (high-frequency) Vs calculated for individual members of the suite of 16 samples of Siletz-Crescent basalts from modal mineralogy and elastic moduli calculated as described in caption to Figure 5. Thick black line shows mean and dotted line shows standard deviation of these values, with temperature. Thin black lines in lower left show a nominal slope of -0.3 m/s/K for purely elastic effects at constant composition, and the best-fit -0.71 ± 0.05 m/s/K for the ensemble. (Right) Anelastic effects estimated for gabbro. Anelasticity is well calibrated only for olivine and olivine-rich rocks, but anelasticity should scale to steady-state creep[13], so anelasticity in gabbro can be estimated by comparing olivine and gabbro viscosities. Grain-boundary diffusion viscosities are probably most relevant given that high-temperature elasticity is accommodated by grain boundary sliding[14]. In gabbros, plagioclase (anorthite) should control aggregate creep properties, and compilations of experimental data show that the dry dislocation creep viscosity for anorthite is higher by a modest factor of 2-5 than olivine for most relevant conditions[15]. Hence, given these similarities and complicating effects of water and grain size, to good approximation the anelasticity of gabbro can be approximated by that of olivine-dominated aggregates[14]. Varying the grain size by an order of magnitude, effective viscosities by a factor of 5 or activation energies by 50 kJ/mol (following approach of Abers et al.[16]) illustrates the uncertainty in this assumption. The colored swath shows the effect of such variations from 100 Monte Carlo perturbations to the olivine anelasticity relationship[14], producing slope changes of 0.1 m/s/K, less than the uncertainty due to H_2O variability. Dotted line shows reference anharmonic variations ignoring both the anelastic and the petrologic effect.*

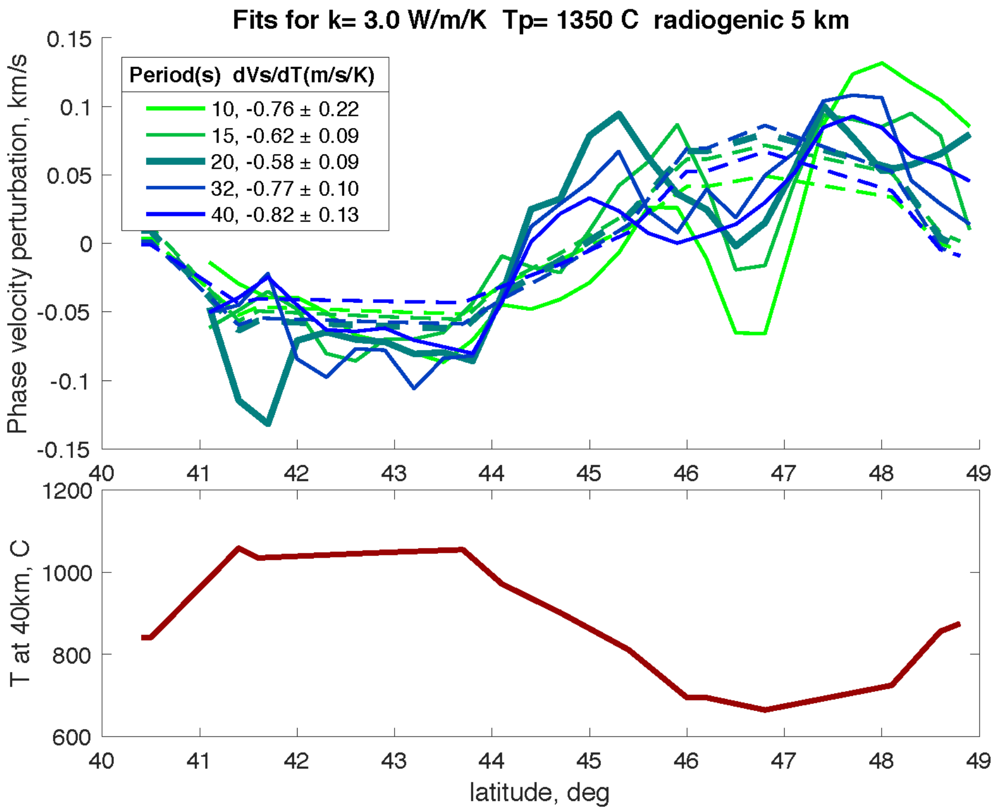

Supplementary Figure 8*. Comparison of observed phase velocity perturbations to predictions from heat flow, based on thermal model shown on Figure S6 (right) and methodology described in text. (top): phase velocity perturbations with latitude, observed (solid) or predicted from heat flow (dashed). Colors correspond to period as shown in legend, along with best-fitting dVs/dT and two-sigma uncertainty. (bottom): predicted temperature at 40 km depth, the approximate Moho. These fits assume thermal conductivity of 3.0 W/m/K, mantle potential temperature of 1350°C and a 5 km thick near-surface layer contributing radiogenic heat of 2 $\mu W/m^3$.*

Bin Center Latitude	*Mono-genetic Volume H07*[a]	*Central Volcano Edifice Volume H07*[a]	*Mono-genetic Volume*[b]	*Central Volcano Edifice Volume*[b]	*Total Quaternary Volcanic Heat, 100% Fractional Crystall-ization*[c]	*Total Quaternary Volcanic Heat, 100% Crustal Melting*[d]	*Total Quaternary Basalt Req., 100% Fractional Crystall-ization*[e]	*Total Quaternar y Basalt Req., 100% Crustal Melting*[f]	*Total Quaternar y Basalt Req., 60% Fractional Crystalliz ation, 40% Crustal Melting*[g]
(°N)	($km^{3)}$	*(km^3)*	*(km^3)*	*SS90 (km^3)*	*(J)*	*(J)*	*(km^3)*	*(km^3)*	*(km^3)*
40	*226*	*265*	*400*	*250*	*$3.04x10^{20}$*	*$2.13x10^{20}$*	*1,314*	*336*	*902*
41	*211*	*1050*	*700*	*325*	*$5.53x10^{20}$*	*$6.63x10^{20}$*	*2,664*	*558*	*1821*
42	*271*				*$1.19x10^{19}$*		*271*	*271*	*271*
43	*261*	*180*	*500*	*160*	*$1.71x10^{20}$*	*$1.96x10^{20}$*	*865*	*363*	*664*
44	*584*	*520*	*1500*		*$4.11x10^{20}$*	*$3.40x10^{20}$*	*2,147*	*761*	*1,592*
45	*176*	*125*	*500*	*20*	*$9.02x10^{19}$*	*$1.53x10^{20}$*	*505*	*256*	*405*
46	*238*	*530*			*$1.54x10^{20}$*	*$4.93x10^{20}$*	*1,018*	*498*	*809*
47	*36*	*200*	*100*	*220*	*$1.56x10^{20}$*	*$2.31x10^{20}$*	*635*	*157*	*443*
48	*8*	*30*		*40*	*$4.11x10^{19}$*	*$2.42x10^{19}$*	*154*	*20*	*100*
49	*25*	*200*		*100*	*$2.18x10^{20}$*	*$1.79x10^{20}$*	*827*	*118*	*543*

[a]Quaternary erupted volumes estimated from Hildreth (2007); [b]Quaternary erupted volumes estimated from Sherrod and Smith (1990); [c]Total Quaternary heat added to crust by observed volcanism using fractional crystallization to produce evolved magmas (see supplementary text); [d]Total Quaternary heat added to crust by observed volcanism using crustal melting to produce evolved magmas (see supplementary text); [e]Total Quaternary basalt required to produce observed volcanism using fractional crystallization to produce evolved magmas; [f]Total Quaternary basalt required to produce observed volcanism using crustal melting to produce evolved magmas; [g]Total Quaternary basalt required to produce observed volcanism using 60% fractional crystallization and 40% crustal melting to produce evolved magmas. See supplementary text for more details on calculations.

***Supplementary Table 1**. Erupted Quaternary volume estimates, calculated heat inputs, and equivalent intruded basalt required.*

Bin Center	10s Phase Velocity		15s Phase Velocity		20s Phase Velocity		32s Phase Velocity	
(°N)	(km/s)	1s	(km/s)	1s	(km/s)	1s	(km/s)	1s
40	3.028	0.046	3.223	0.071	3.374	0.021	3.693	0.015
41	3.077	0.067	3.191	0.065	3.375	0.022	3.644	0.018
42	3.074	0.038	3.195	0.046	3.327	0.017	3.616	0.015
43	3.047	0.033	3.174	0.043	3.343	0.010	3.588	0.009
44	3.059	0.046	3.214	0.047	3.364	0.012	3.618	0.01
45	3.089	0.045	3.246	0.045	3.48	0.009	3.718	0.008
46	3.137	0.039	3.305	0.036	3.459	0.009	3.7	0.007
47	3.097	0.055	3.263	0.045	3.447	0.009	3.729	0.006
48	3.239	0.044	3.336	0.034	3.484	0.011	3.76	0.007
49	3.208	0.057	3.265	0.068	3.499	0.006	3.694	0.006

	32s Phase Velocity		40s Phase Velocity		50s Phase Velocity		Av. Heat Flow
(°N)	(km/s)	1s	(km/s)	1s	(km/s)	1s	(mW/m^2)
40	3.693	0.015	3.727	0.014	3.829	0.019	62.3
41	3.644	0.018	3.726	0.013	3.782	0.018	83.9
42	3.616	0.015	3.728	0.012	3.790	0.017	86.5
43	3.588	0.009	3.704	0.009	3.800	0.010	86.7
44	3.618	0.010	3.713	0.010	3.817	0.013	83.6
45	3.718	0.008	3.794	0.008	3.880	0.009	74.9
46	3.700	0.007	3.777	0.008	3.864	0.007	57.8
47	3.729	0.006	3.808	0.007	3.897	0.007	58.7
48	3.760	0.007	3.851	0.008	3.943	0.009	62.1
49	3.694	0.006	3.821	0.006	3.922	0.008	75.5

Supplementary Table 2. *Phase velocities and Average Measured Heat Flow used in this study. In order to quantitatively compare heat flow to seismic velocities and the calculated magmatic heat budgets the heat flow values along the arc were extracted from the contoured Cascades heat flow map of Ingebritsen and Mariner[17] (their Fig. 5), based in turn on the the USGS heat flow database[18]. To convert the contoured heat flow map to values at the arc axis, the arc was divided into 0.25° slices of latitude between 39.5 and 49.5°N, and the average heat flow was estimated by visually determining four heat flow values per slice (representing bins of 0.45°) between 120.5 and 122.3°W longitude, the same longitudinal swath for the reported seismic velocities. From these we determined an average value per 0.25° latitudinal slice and an average value per 1° latitude bin.*

	10 s phase velocity	15 s phase velocity	20 s phase velocity	32 s phase velocity	40 s phase velocity	60 s phase velocity	Average Surface Heat Flow
15 s phase velocity	0.81						
20 s phase velocity	0.78	0.84					
32 s phase velocity	0.63	0.87	0.85				
40 s phase velocity	0.87	0.87	0.92	0.88			
60 s phase velocity	0.82	0.88	0.92	0.86	-0.79		
Average Surface Heat Flow	-0.38	-0.79	-0.61	-0.84	-0.62	-0.66	
Monogenetic Volume	-0.64	-0.54	-0.66	-0.70	-0.78	-0.69	0.50
Main Edifice Volume	-0.32	-0.41	-0.44	-0.44	-0.52	-0.67	0.34
Total Quaternary Volcanic Heat, 100% Fractional Crystallization	-0.37	-0.43	-0.31	-0.34	-0.49	-0.46	0.27
Total Quaternary Volcanic Heat, 100% Crustal Melting	-0.35	-0.35	-0.38	-0.41	-0.50	-0.65	0.23
Total Quaternary Basalt Required, 100% Fractional Crystallization	-0.43	-0.45	-0.38	-0.42	-0.57	-0.56	0.32
Total Quaternary Basalt Required, 100% Crustal Melting	-0.59	-0.48	-0.54	-0.62	-0.75	-0.70	0.39
Total Quaternary Basalt Required, 60% Fractional Crystallization, 40% Crustal Melting	-0.46	-0.47	-0.42	-0.47	-0.61	-0.59	0.34

[red box] $P \leq 0.05$

***Supplementary Table 3**. Summary of Pearson correlation coefficient and significance for seismic velocities, average heat flow and the calculated volcanic volumes and heat fluxes for our latitude bins along arc. Cells display the correlation coefficient (r) and are color coded (see legend) by the P value calculated for available degrees of freedom, which represents the probability that the observed correlation occurs due to chance. Cells in red have less than a 5%*

probability of the apparent correlation being due to chance. Volumes used are from Hildreth[1]. *See Supplementary Table S1 and S2 for the values used.*

References Cited

1. Hildreth, W. (2007). Quaternary magmatism in the Cascades – geologic perspectives. Professional Papers of the United States Geological Survey, 1744.
2. Sherrod, D.R., and Smith, J.G. (1990) Quaternary Extrusion Rates of the Cascade Range, Northwestern United States and Southern British Columbia: J. Geophys. Res., 95, 19465–19474.
3. Donnelly-Nolan, J.M., Grove, T.L., Lanphere, M.A., Champion, D.E., and Ramsey, D.W. (2008) Eruptive history and tectonic setting of Medicine Lake Volcano, a large rear-arc volcano in the southern Cascades: Journal of Volcanology and Geothermal Research, 177, 313–328, doi: 10.1016/j.jvolgeores.2008.04.023.
4. Grove, T.L., Gerlach, D.C., and Sando, T.W. (1982) Origin of Calc-Alkaline Series Lavas at Medicine Lake Volcano by Fractionation, Assimilation and Mixing: Contributions to Mineralogy and Petrology, 80, 160–182.
5. Blatter, D.L., Sisson, T.W., and Hankins, W.B., (2013) Crystallization of oxidized, moderately hydrous arc basalt at mid- to lower-crustal pressures: implications for andesite genesis: Contributions to Mineralogy and Petrology, 166, 861–886, doi: 10.1007/s00410-013-0920-3.
6. Mandler, B.E., Donnelly-Nolan, J.M., and Grove, T.L. (2014) Straddling the tholeiitic/calc-alkaline transition: the effects of modest amounts of water on magmatic differentiation at Newberry Volcano, Oregon: Contrib. Mineral. Petrol., 168, 1–25, doi: 10.1007/s00410-014-1066-7
7. Grove, T.L., Elkins-Tanton, L.T., Parman, S., Chatterjee, N., Muentener, O., and Gaetani, G.A. (2003) Fractional crystallization and mantle-melting controls on calc-alkaline differentiation trends. Contributions to Mineralogy and Petrology, 145, 515–533.
8. Marsh, B. D. (1981) On the crystallinity, probability of occurrence, and rheology of lava and magma. Contrib. Mineral. Petrol. 78, 85–98.
9. Janiszewski, H. A. (2017), New insights on the structure of the Cascadia subduction zone from amphibious seismic data, PhD thesis, 148 pp, Columbia Univ., New York.
10. Calkins, J. A., G. A. Abers, G. Ekström, K. C. Creager, and S. Rondenay (2011), Shallow structure of the Cascadia subduction zone beneath western Washington from spectral ambient noise correlation, J. Geophys. Res., 116, B07302, doi:10.1029/2010JB007657.
11. Herrmann, R. B., and C. J. Ammon (2004), Computer Programs in Seismology: Surface Waves, Receiver Functions, and Crustal Structure, version 3.30, St. Louis Univ., Saint Louis, Mo.
12. Herrmann, R. B. (2013), Computer programs in seismology: An evolving tool for instruction and research, Seismol. Res. Lett., 84(6), 1081– 1088, doi:10.1785/0220110096.
13. McCarthy, C., Y. Takei, and T. Hiraga (2011), Experimental study of attenuation and dispersion over a broad frequency range: 2. The universal scaling of polycrystalline materials, J. Geophys. Res., 116, art. no. B09207, doi:09210.01029/02011JB008384.

14. Jackson, I., and U. H. Faul (2010), Grainsize-sensitive viscoelastic relaxation in olivine: Toward a robust laboratory-based model for seismological application, Phys. Earth Planet. Int., 183, 151-163.
15. Burgmann, R., and G. Dresen (2008), Rheology of the lower crust and upper mantle: evidence from rock mechanics, geodesy, and field observations, Ann. Rev. Earth Planet. Sci., 36, 531-567.
16. Abers, G. A., K. M. Fischer, G. Hirth, D. A. Wiens, T. Plank, B. K. Holtzman, C. McCarthy, and E. Gazel (2014), Reconciling mantle attenuation-temperature relationships from seismology, petrology and laboratory measurements, Geochem. Geophys. Geosys. (G3), 15, 3521-3542, doi:10.1002/2014GC005444.
17. Ingebritsen, S.E. & Mariner, R.H. (2010) Hydrothermal heat discharge in the Cascade Range, northwestern United States. Journal of Volcanology and Geothermal Research 196, 208–218.
18. Williams, C.F., DeAngelo, J. (2008) Mapping geothermal potential in the western United States. Transactions of the Geothermal Resources Council 32, 181–188.
